# From the origins to the stream of consciousness and its neural correlates

**DOI:** 10.3389/fnint.2022.928978

**Published:** 2022-11-04

**Authors:** Sergey B. Yurchenko

**Affiliations:** Independent Research Center of Brain and Consciousness, Andijan, Uzbekistan

**Keywords:** stream of consciousness, volition, criticality, complexity, brain dynamics, quantum, evolution

## Abstract

There are now dozens of very different theories of consciousness, each somehow contributing to our understanding of its nature. The science of consciousness needs therefore not new theories but a general framework integrating insights from those, yet not making it a still-born “Frankenstein” theory. First, the framework must operate explicitly on the stream of consciousness, not on its static description. Second, this dynamical account must also be put on the evolutionary timeline to explain the origins of consciousness. The Cognitive Evolution Theory (CET), outlined here, proposes such a framework. This starts with the assumption that brains have primarily evolved as volitional subsystems of organisms, inherited from primitive (fast and random) reflexes of simplest neural networks, only then resembling error-minimizing prediction machines. CET adopts the tools of critical dynamics to account for metastability, scale-free avalanches, and self-organization which are all intrinsic to brain dynamics. This formalizes the stream of consciousness as a discrete (transitive, irreflexive) chain of momentary states derived from critical brain dynamics at points of phase transitions and mapped then onto a state space as neural correlates of a particular conscious state. The continuous/discrete dichotomy appears naturally between the brain dynamics at the causal level and conscious states at the phenomenal level, each volitionally triggered from arousal centers of the brainstem and cognitively modulated by thalamocortical systems. Their objective observables can be entropy-based complexity measures, reflecting the transient level or quantity of consciousness at that moment.

## Introduction

What can be said with certainty about the brain is that this is a complex dynamical system: (i) governed by the deterministic laws of nature at the physical (causal) or “hard” level which (ii) implements cognitive processing at the computational (unconscious) or “soft” level while (iii) its conscious manifestations occur at the phenomenal (mental) or “psyche” level. These three levels are also separated across different spatial-temporal scales. Neural activity is presented at the microscale of cellular interactions, cognitive processing occurs at the mesoscale of neural populations, and conscious experience emerges only at the macroscale of brain dynamics (Varela, 1995; Revonsuo and Newman, 1999). Another way to distinguish between the hard and the soft level in terms of network neuroscience is to relate the former to structural (anatomical) connectivity of hard-wired neurons, where causation really occurs. In contrast, the soft level corresponds to functional connectivity, where distant correlations between brain regions take place. Computations at the soft level cannot violate causal interactions at the hard level but are merely imposed upon physically interacting neurons. They cannot change brain dynamics that obey deterministic laws.

Thus, the mind-brain duality can be viewed in the formal terms of “hard/soft” parallelism between causation and computation. While computation is typically defined through symbolic manipulations of information, its processing depends ultimately on causal chains between inputs and outputs. The question of how those symbolic manipulations translate into conscious experience in the brain and why other information-processing systems such as computers and AI systems lack conscious experience at the psyche level is one of the most mysterious problems in neuroscience.

The main postulate of Gestalt psychology is that conscious experience is a unified whole, which is greater than the sum of its parts. More generally, this postulate is known in the context of the spontaneous emergence of unexpected higher-level phenomena that are not reducible to their low-level constituents (Bedau and Humphreys, 2008; Gibb et al., 2019). A related issue in neuroscience is specified as the binding problem: how brain regions composed of billions of (unconscious) neurons can generate a unified conscious experience at a given moment of time (Edelman, 2003). In fact, many (if not all) theories of consciousness originate from or, at least, can be reduced to how they decide this problem. Among the candidates proposed by known theories are integrated information (II) of irreducible causal mechanisms (Tononi, 2008), global workspace (GW) for broadcasting (Baars et al., 2013; Mashour et al., 2020), updating of priors (UP) in predictive processing (Knill and Pouget, 2004; Clark, 2013), meta-representation (MR) by recurrent processing (Lamme, 2006; Rosenthal, 2008), self-organized criticality (SOC) in brain dynamics (Werner, 2009; Kozma and Freeman, 2017), adaptive resonance (AR) of brain structures (Grossberg, 2017; Hunt and Schooler, 2019), and even large-scale quantum entanglement with a consequent collapse (Hameroff and Penrose, 2014; Fisher, 2015).

Those in turn can be grouped by the similarity of mechanisms or processes involved: II + GW by integration-differentiation processes, GW + UP + MR by feedback mechanisms, SOC + AR by spontaneous synchronization and phase transition. However, any grouping is somewhat arbitrary as the underlying mechanisms can converge to a thermodynamic account: the neural binding arises when brain activity is balanced on the edge between order and disorder. Broadly speaking, consciousness emerges in a very special state of matter somewhere between a perfect crystal and an ideal gas (Tegmark, 2015).

The cognitive evolution theory or CET (Yurchenko, 2022), outlined here, adopts the SOC approach for its apparent advantages over the above models in studying consciousness. SOC is neurophysiologically reliable in resolving the binding problem without resorting to exotic physics or mysterious mind-matter dualism. This provides rich mathematical formalisms applicable to brain dynamics. SOC also proposes the avenue for explaining universal dynamical capacities of the brain to account for large-scale emergent phenomena without involving the so-called downward or top-down causation that might make consciousness like a homunculus due to “synergistic emergence” (Lau, 2009; Hoel et al., 2013; Mediano et al., 2022). On the other hand, SOC is abundantly presented in nature (Bak, 1996; Jensen, 1998; Haken, 2004). However, we do not normally assume that an arbitrary physical system exhibiting critical signatures is conscious. Something else must be inherent to a system to generate consciousness.

CET starts from the obvious fact that the only place where consciousness certainly resides is the brain. There are four principled features that make the brain distinct from all other critical systems. First, the brain consists of neurons specialized for transmitting information over spike patterns. The neurons had evolved from autonomous biological cells possessing all properties of life not merely as mechanistic binary devices. Hence, consciousness is a property of living systems. Second, there are arousal mechanisms regulating sleep-wake cycles in these living systems which can be suppressed by anesthetics. It is also known that damage to arousal nuclei causes immediate coma when the rest of the brain can remain intact (Parvizi and Damasio, 2001; Giacino et al., 2014). Hence, consciousness is impossible without involving special neural nuclei in the brain responsible for arousal. Third, the brain learns and accumulates knowledge. Hence, the brain is a cognitively evolving system. However, AI systems can learn and even cognitively evolve without any kind of awareness. Is there something else that is inherent to the conscious brain but absent in unconscious machines?

The fourth and ultimate distinction is volition, the ability to make free decisions not causally predetermined from the past. This valuable property is thought to be intrinsic to many (if not all) brain systems regardless of their conscious features. In contrast, we do not normally grant volition to computers and AI systems even if they can sometimes surpass humans in cognitive performance. The volition of this kind is akin to one that can be ascribed to clockwork’s engine, i.e., it is an ordinary physical process carrying energy out of one place into another. How much our intuition is right by assuming that consciousness and volition are evolutionarily linked?

With the advent of causation neuroscience, the detailed relationship between statistical models of neural activity and actual causation in the brain is intensively debated (e.g., Albantakis et al., 2019; Reid et al., 2019; Weichwald and Peters, 2021). Statistical measures such as Granger Causality (Granger, 1969) or Entropy Transfer (Schreiber, 2000) had been suggested to infer some aspect of causal interaction among neural entities that are then modeled on a particular structural time-directed graph over a set of nodes. Their definition is based entirely on the predictability of some time-series. If Y contains information that helps to predict X beyond the degree to which X already predicts its own future, then Y is said to have a causal influence on X. Causal modeling must reflect dynamic processes irrelevant to the question of whether a system of interest processes information or not. In cognitive neuroscience, causal inference is based on a synthesis of functional and effective connectivity extracted from neuroimaging data (Friston, 1994). The “gold standard” to establishing whether a stimulus variable Y affects the target variable X is to introduce controlled perturbations to the brain.

It must be however emphasized that causality measures do not necessarily reflect physical causal chains (Seth, 2008). Meanwhile, brain dynamics are commonly believed to evolve completely in causal ways over conscious and unconscious volitional repertoires of the brain. Within those volitional repertoires, the ability generally labeled “free will” is associated with the sum of executive functions, self-control, decision-making, and long-term planning. Thus, it makes free will inseparable from the biological function of consciousness, evolution-driven, and implicitly active (Feinberg and Mallatt, 2020). Putting the above question differently, could consciousness supervene on its own physical substrate to choose the course of action free of predetermination from the past?

To answer this question, a unified theory of consciousness should account for brain activity at three hierarchical levels: (i) at the causal (hard) level; (ii) at the computational (soft) level; and (iii) at the phenomenal (psyche) level. The first two levels should explain how subjective experience and self-awareness emerge from the underlying brain dynamics over which cognitive processing is carried out.

## Consciousness and Volition in Brain Dynamics

The stochastic account of free volition can be often found in the literature. For example, Rolls (2012) suggests: “in so far as the brain operates with some degree of randomness due to the statistical fluctuations produced by the random spiking times of neurons, brain function is to some extent non-deterministic, as defined in terms of these statistical fluctuations.” Since the brain contains billions of neurons, causal processes can be only estimated with the help of network statistics extracted from different neuroimaging data. However, probabilistic (counterfactual) descriptions, as those derived from causality measures, reflect the state of our knowledge (ignorance) that, by itself, does not violate determinism. Brain dynamics can still be completely deterministic, i.e., predetermined.

Many free-will advocates usually suggest that if even conscious volition cannot violate determinism at the causal level, it can still be involved in long-term planning of actions at the psyche level. They argue that the ability to use optimal algorithms in predictive processing would be a much more important factor than whether the brain operates deterministically or not (Rolls, 2020). Does it mean that by using those algorithms AI systems might suddenly acquire free volition? Relevantly, relying on this pure computational aspect of volition in the context of the hard-soft duality would imply no obstacle in creating machine (hence, copyable) consciousness at the psyche level (Dehaene et al., 2017; VanRullen and Kanai, 2021). This comes from the observation that there can be no operational difference between a perfect computational simulation of, for instance, Alice’s actions and an *in silico* copy of her consciousness running automatically on many digital clones (Aaronson, 2016). Thus, ignoring the hard-soft duality also entails the problem of the privacy of consciousness: the clones should know what it is like to be Alice.

A typical scenario suggested for manifesting free volition at the computational (soft) level is one where Alice can consciously plan something ahead of time, for example, visiting her friends tonight. Thus, Alice’s freedom to choose can be proven by achieving her goal. Upon a closer examination, all such scenarios are behavior-driven, yet based on uncertain and rough assumptions about what occurs at the microscale of neural interactions. Within a rigorous physical framework, the spatial and temporal locations of action should be specified *via* the stream of conscious states, each processed by the brain at the hard level.

Suppose Alice plans to visit her friends at the moment *t*_0_ when her consciousness is in a state *X*. Whenever she could reach this goal, her conscious state at that exact moment *t* would be *Y*. But the state *Y* should in turn have been consistently processed from the previous state *Y* − 1 over ubiquitous causal chains. How could it be done freely? Moreover, the manifestation of the conscious will should be related not to Alice being at her goal state *Y* but to its mental initiation in *X*. Indeed, after the decision has been made, her goal-directed behavior could be completely deterministic. However, this state should also be causally processed from the previous state *X* − 1, and so on. How could mental initiation be free of the past? Hence, if consciousness cannot choose the next state from a given past state, no future state in the stream can be chosen at all.

On the other hand, if the brain cannot make a choice free of the past, the old-fashioned fatalism, also known as superdeterminism in the context of quantum mechanics (‘t Hooft, 2016; Hossenfelder and Palmer, 2020), would prevail. This states that neither consciousness nor even the brain might violate deterministic laws to do otherwise than what has been predetermined by the past. How could free volition be reconciled with computational models of consciousness at the hard level?

## Volition in Theories of Consciousness

Consciousness and volition are intrinsically linked, and both are largely ignored in neuroscience. Although there are now a plethora of various theories of consciousness, the free will problem still remains largely neglected. The theories do not explain how the brain integrates consciousness (psyche), cognition (soft), and volition (hard) seamlessly across the three hierarchical levels. Yet, as being static in nature, most of them aim to explain the structure of conscious experience *per se* without accounting for the successive alternation of conscious states over time. Many authors attempt to compare these theories (Doerig et al., 2021; Del Pin et al., 2021; Signorelli et al., 2021), or even to reconcile some of them (Shea and Frith, 2019; Chang et al., 2020; Graziano et al., 2020; Mashour et al., 2020; Northoff and Lamme, 2020; Mallatt, 2021; Seth and Hohwy, 2021; VanRullen and Kanai, 2021; Niikawa et al., 2022). Another tendency is to incorporate these static theories into a more general dynamical framework such as the Temporo-spatial Theory of Consciousness (Northoff and Zilio, 2022) which is somewhat reminiscent of Operational Architectonics (Fingelkurts et al., 2010), the whole-brain mechanistic models from a bottom-up perspective (Cofré et al., 2020), or the self-organizing harmonic modes coupled with the free energy principle (Safron, 2020).

In general, all these theories are not concerned with brain dynamics at the underlying hard level from which conscious states emerge. As being static in design, they have also missed another fundamental aspect of consciousness, namely, its cognitive evolution over a lifetime going by accumulating new knowledge and skills. Accordingly, the stream of consciousness (though implied) is not properly defined. At the same time, they all ascribe a special, active role to consciousness while being indifferent to the free will problem by adopting the view that consciousness can somehow influence brain dynamics at the psyche level. It is implicitly assumed that consciousness: (i) facilitates learning; (ii) is useful for voluntary decision-making; and (iii) provides stability to the objects of experience in an egocentric framework (Seth and Baars, 2005).

For example, Integrated Information Theory (IIT) defines consciousness as information a system is able to integrate. It does not consider perception, cognition, and action at all by aiming at the quantitative account of phenomenal consciousness due to “irreducible causal mechanisms” (Oizumi et al., 2014). It makes the striking conclusion that “a conscious choice is freer, the more it is causally constrained within a maximally irreducible complex” (Tononi, 2013) without explaining how free volition might be manifested there. Such a kind of volition turns out to be just superdeterministic (see below). It could well account for the individuality of subjective experience as specified by the complex at the psyche level but not for the indeterminism of volition at the hard level. The IIT approach can be even extended to a general idea that the very evolution of life is just the triumph of determinism. It argues that living systems can thrive across the universe as soon as these autonomous systems have more cause-effect power than non-living environments (Marshall et al., 2017).

On the contrary, Predictive Processing Theory (PPT) can well explain perception, cognition, and action by making the brain an error-minimizing machine (Clark, 2013; Seth and Hohwy, 2021). Although PPT is unclear about where exactly, between priors and posteriors, the conscious experience should appear, it can in principle separate discrete conscious states emerging at the psyche level as ultimate decisions of Bayesian learning from unconscious predictive processing at the soft level. Nevertheless, PPT cannot still account for free volition, which is covertly embedded in attentional effort and active inference (Friston et al., 2013; Pezzulo et al., 2018). This is just the point where free will and active consciousness converge. If consciousness is an algorithm for the maximization of resilience (Rudrauf et al., 2017), then PPT has to explain how and why conscious processing in the brain should differ from deep machine learning in AI systems, which can exploit the same computational models but lack both volition and conscious experience.

Another dominant theory, Global Workspace Theory (GWT), relies explicitly on the active role of consciousness at the psyche level, which is required for global access, broadcasting information, and self-monitoring (Dehaene and Changeux, 2011; Baars et al., 2013). According to the theory, a physical system “whose successive states unfold according to a deterministic rule can still be described as having free will, if it is able to represent a goal and to estimate the outcomes of its actions before initiating them” (Dehaene and Naccache, 2001). Thus, GWT adopts just the aforementioned scenario with Alice deciding where she will be tonight. It is therefore not surprising that GWT does not suggest any obstacle to machine consciousness (Dehaene et al., 2017), which by virtue of its cognitive architecture could be spontaneously endowed with free will.

Finally, psychological theories such as Higher-Order Thought (Lau and Rosenthal, 2011), Attention Schema (Graziano et al., 2020), Radical Plasticity Thesis (Cleeremans, 2011), or Self Comes to Mind (Damasio, 2010) argue that self-awareness or metacognition would separate conscious states from unconscious processing *via* self-referential mechanisms. These mechanisms would make the brain aware of its own states, unlike other biological and artificial networks. Accordingly, conscious will in these theories is similar to “conscious veto” suggested by Libet (1985) to circumvent the findings of his famous free will experiments.

The Libet-type experiments have been based on two temporal measures: the readiness potential detected from the supplementary motor area, and the awareness of wanting to move reported with the clock. The delay observed between neural motor predictors at the hard level and conscious intentions at the psyche level was around several hundred milliseconds (Libet, 1985; Guggisberg and Mottaz, 2013; Schultze-Kraft et al., 2016), thereby making conscious intentions a post-factum phenomenon. Libet had proposed that it could occur due to conscious deliberations before consciousness could block an action with the explicit veto on the movement. Nevertheless, since any kind of intentional veto must also be causally processed, it has been noted that the blocking itself could be predetermined (Soon et al., 2013).

Yet, some authors assume that noisy neural fluctuations can be involved in self-initiated actions (Schurger et al., 2012). They argue that the key precursor process for triggering internally-generated actions could be essentially random in a stochastic framework (Khalighinejad et al., 2018). First, those actions generated internally by the brain from noisy fluctuations would have little relevance to the ability of consciousness to make its own choice. Meanwhile, in the psychological theories mentioned above, conscious will would involve higher-order thoughts to check many moves ahead of time, and then with this information to make a free choice (Rolls, 2020). Second, stochastic noise as being classical (thermal) in nature does not violate determinism to account for freely generated actions even on the brain’s authorship. Something else is needed.

In contrast, quantum-inspired theories of consciousness take seriously the free will problem by adopting quantum indeterminism that might affect brain dynamics at the hard level. They are based on the conceptualization of free will suggested by Bell in his famous theorem (Bell, 1993). The key assumption of Bell was that Alice’s free choice (as defined above) should not be controlled by any hidden (unknown to our modern knowledge) deterministic variables *λ*. For example, these variables might include all the internal and external information about the past of Alice’s brain and everything in her environment. The choice is then formalized by conditional probability,


(1) p(A|λ)=p(A)

The theorem had shown that under measurements of preciously prepared quantum experiments no such variables should exist in principle unless we had to agree on a “cosmic conspiracy” that constrained Alice to make not any choice but just the one that did not violate standard statistical correlations of the Bell’s inequality (Gallicchio et al., 2014). Note that, as suggested beyond the neuroscientific context of Libet-type experiments, Equation (1) does not discriminate between a choice made by Alice with her conscious will, and a choice internally generated by her brain itself. The only thing required there is that *A* has not been predetermined from the past by *λ*. Thus, by ruling out conscious will from Libet-type experiments, only the indeterminism of neural activity can account for the Bell theorem, which has been well-confirmed experimentally (e.g., Aspect et al., 1982).

Orchestrated Objective Reduction (Orch OR) of Hameroff and Penrose (2014), the most known of quantum-inspired theories of consciousness, is explicitly based on the indeterminism of the wavefunction collapse or “objective reduction” (OR), also known as the measurement problem. In quantum mechanics, the measurement problem has the striking property of observer-dependence. In contrast, Penrose argues for a real quantum-gravitational OR that occurs everywhere in the universe, independently of observation, as spontaneous “proto-conscious” events that are then orchestrated in microtubules of the brain to give rise to consciousness and volition. According to Orch OR, consciousness must be in principle incomputable as being orchestrated (Orch) by quantum entanglement with consequent OR. Nonlocal correlations between microtubules (for global binding of dissociated brain regions) and backward time referral (for closed causal loops) are then required “to rescue causal agency and conscious free will” (Hameroff, 2012).

Remarkably, the mathematicians Conway and Kochen in their Free Will Theorem (a modified version of the Bell theorem) make a statement very similar to that of Penrose (though without concerning themselves with OR): “If conscious observers have a certain freedom to choose, then particles already have their own share of this freedom” (Conway and Kochen, 2008). It is often argued that the quantum randomness on which the statement reposes has little to do with “free will” thought to be caused by a reason rather than by chance (Koch, 2009; Aaronson, 2016). Conway and Kochen had however noted that if a subject’s action was indeed free of the past, then it should be difficult to find a testable (i.e., objective from a third-person perspective) difference between physical randomness and the subject’s genuine behavioral freedom, both not being predetermined by the previous history of the universe. In the context of neuroscience, their statement has to be inverted and specified as follows:

*If particles have some freedom to respond to the environment independently of the past, then the observers have the same kind of initial freedom due to a quantum mechanism Bell-certified by Equation (1) in their brains*.

Of course, ascribing free will to living or non-living systems does not depend anyhow on quantum effects. Volition is typically associated with the action a system is capable of initiating. It necessarily involves causation. However, what is the sense of saying that a clockwork toy has the volition to move, or, that a more sophisticated AI system is self-initiated in performing a cognitive task? To discriminate between volition and trivial energy flow in an arbitrary (natural or artificial) system the former must not be predetermined from the past. Volition is not a matter of consciousness or cognition but a matter of causal freedom. Under this definition, quantum particles can have some sort of freedom but this freedom is washed out in macroscopic systems.

To bind volition with consciousness, we need to translate physical predetermination into the notion of predictability. First of all, deterministic systems can in principle be perfectly predictable. It is already a matter of our current knowledge and technology. Now let us modify the famous Turing test where a machine might mimic a human by answering various questions. Suppose, a future machine can be trained to predict precisely Alice’s every choice ahead of time. The machine can thus perfectly simulate Alice’s stream of consciousness so that we might be fooled by asking any question. Why then should we deny that the machine possesses a copy of Alice’s consciousness and, thus, is itself conscious?

Hence, if we disagree that humans are very sophisticated but still deterministic and copyable machines, there must be something that prevents one from making a deterministic machine conscious. This is volition. Indeed, if Alice’s brain is able to make a choice not predetermined from the past, there cannot be in principle a perfect predictor of Alice’s stream of consciousness. This makes her consciousness unique, i.e., non-copyable for any future technology. Thus, the reason to assume the above statement stems fundamentally from the evolutionary perspective.

CET suggests a dynamical model based on a framework, drawn over diverse neuroscientific domains, with contributions from critical dynamics, predictive processing, and evolutionary neurobiology to approach a unified theory of consciousness grounded in physics. The widespread idea that brains are error-minimizing machines has neglected a crucial ingredient of its adaptive framework: before minimizing an error the brain should already have that error internally triggered under cognitive correction. CET argues that the brain can be viewed more generally as both an error-amplifier and a modulator of the primitive volitional reflexes based on chemo- and photoreceptors of unicellular organisms. In doing so, CET makes the assumption that brains have primarily evolved as volitional (quantum in origin) subsystems of organisms at the hard level. CET emphasizes the importance of randomness in evolution (Yurchenko, 2021a) contrary to the idea that life can be viewed as the triumph of determinism (Marshall et al., 2017).

## Volition from The Evolutionary Perspective

In modern science of consciousness, the Cartesian presumption that animals are only biological automata incapable of experiencing conscious and emotional states looks chauvinistic or even perverse (Lamme, 2018; Fields et al., 2021). Nonetheless, the tradition to separate humans from the rest of the animal kingdom is still persistent. Now the division has shifted to the free will debate. It is assumed that only humans enjoy free will due to the sophisticated computations the human brain is able to carry out at the soft level, while other animals, though passively conscious, are deprived of this gift (Doyle, 2009). CET finds this division evolutionarily implausible.

Maintaining homeostasis in the face of dangerous and unfortunate environmental conditions is the basic imperative for the evolution of the brain (Ashby, 1960). The only valuable advantage the living systems might gain over non-living systems is the ability to decide their way freely among stimulus-reaction repertoires. This ability is commonly referred to as volition. Meanwhile, volitional mechanisms of the brain are still ignored in cognitive neuroscience, remaining hidden under attentional effort, enactive cognitive function, and conscious control. CET argues that volition is a key neural mechanism of evolution placed between organisms and non-living systems. Life could not have flourished on Earth without volitional mechanisms which are the only neural candidate that opens a door to the stimulus-reaction repertoires over the “tyranny” of causal chains.

The underlying idea here is the one of evolutionary neurobiology: the ultimate aim of brain evolution over species, from simplest organisms to humans, was to maximize their adaptive success. Under selection pressure, the main function of neural systems resulted in their ability to integrate and process a very large number of sensory inputs and motor responses occurring in parallel (Edelman, 2003). Thus, the cognitive evolution of the brain over every organism’s lifetime (ontogeny) and the general evolution of the brain over species (phylogeny) should go hand in hand to promote each other. CET makes the general assumption that after acquiring free volition mechanisms to overcome the fatalism of cause-effect interactions which govern completely non-living systems, organisms could have advanced their adaptive success only by evolving cognitive functions capable of predicting future events. Cognition and memory should have evolved exclusively as adaptive (computational) abilities of organisms at the soft level to benefit from some underlying volitional mechanism at the hard level. Otherwise, evolution would have had no reason to advance those adaptive properties over rigid causal chains in deterministic systems.

How much is it plausible that evolution had endowed primitive networks of simplest organisms with some kind of freedom which would have been of value for them in survival and reproduction? Can invertebrates have some volitional mechanism, evolutionarily embedded in their neural networks to make a choice not predetermined by the past? CET argues that primitive neural networks should have primarily evolved as free-volitional subsystems of organisms, not as deterministic prediction machines (Knill and Pouget, 2004; Clark, 2013), requiring larger biological resources. Accordingly, their conscious properties, typically related to higher animals, should have appeared later than their unconscious cognitive functions presented in invertebrates (Brembs, 2011). Instead of being an active participant that creates an internal representation of the environment, the conscious experience would be an extension of primitive volitional-emotional motivations (Mashour and Alkire, 2013). Thus, contrary to the idea that evolution had evolved consciousness to perform complex volitional actions (Pennartz et al., 2019; Feinberg and Mallatt, 2020), in CET consciousness is a byproduct of both volitional mechanisms and unconscious cognitive processing grounded in sensorimotor coupling (Engel et al., 2013). With this assumption on the origins of consciousness, CET suggests a radically new and physically rigorous solution to the free will problem.

Unlike the known quantum-inspired models involving the most mysterious quantum-mechanical effects to account for quantum computing and/or quantum memory storage that might directly mediate consciousness (Hameroff and Penrose, 2014; Fisher, 2015; Georgiev, 2020), CET suggests a minimal use of quantum randomness at the sub-cellular level. This refers to the most reliable mechanisms like the Beck-Eccles quantum trigger, proposed as a quantum-mechanical model for the mechanism of exocytosis (Beck and Eccles, 1992, 1998). Exocytosis is a discrete all-or-nothing event consisting of the opening of presynaptic membrane channels with the release of neurotransmitters in the synaptic cleft. The trigger is based on the tunneling of a quasi-particle across a potential energy barrier. Beck and Eccles (1992) argue that “the mental intention (the volition) becomes neurally effective by momentarily increasing the probability of exocytosis in selected cortical areas such as the supplementary motor area.” Thus, they maintain the cortex-centered conceptualization of *conscious* free will in Libet-type experiments.

Instead, CET places the mechanism in the brainstem to account for the indeterminism of unconscious free volition initiated in the arousal nuclei. This key quantized event could then be physically amplified across many spatiotemporal scales due to neuronal avalanches which are intrinsic to SOC (Blanchard et al., 2000; Beggs and Plenz, 2003; Chialvo, 2010; Tognoli and Kelso, 2014). The brainstem is a phylogenetically ancient brain region that comprises various nuclei executing many vital autonomic functions for maintaining homeostasis such as blood pressure, heartbeat, or blood sugar levels (Paus, 2000; Parvizi and Damasio, 2001). Those autonomic functions should have evolved much early than thalamocortical regions engaged in elaborating cognitive contents and conscious experience.

Remarkably, this ancient region also contains the arousal centers responsible for permanent vigilance upon which the stream of consciousness reposes. Its arousal machinery is a precondition for behavior and conscious experience (Mashour and Alkire, 2013). CET proposes that each conscious state in the stream is volitionally driven at the causal (hard) level from subcortical arousal centers *via* the ascending reticular activating system (ARAS) to thalamocortical systems, involved in perception and cognition at the computational (soft) level. In CET, conscious experience emerges at the psyche level as a passive phenomenon of cognitive brain evolution that goes by accumulating new knowledge and skills freshly updated in memory networks.

By postulating the quantum mechanism that will be called the “neurophysiological free-volition mechanism” (NFVM) and placed into the brainstem, CET can account for the indeterminism of brain dynamics without resorting to large-scale quantum mysteries^1^. The corollaries are the following. While brain dynamics will still be presented by classical stochastic processes as those traditionally described in standard models of neuroscience, the stream of consciousness will no longer be predetermined from the past by hidden deterministic variables *λ* due to the presence of the NFVM. This makes Alice’s consciousness (whose secured privacy is guaranteed by the Bell-certified NFVM) unique but gives her consciousness no power over the brain unlike typical scenarios mentioned above.

In general, the controversy around free will has been inherited by the science of consciousness from the mind-body problem, originally discussed in philosophy. What exactly should be associated with Alice? Is it her brain or her consciousness generated by her brain (leaving aside her body that makes the brain alive and functional)? In CET, if Alice is associated with her brain, she has free volition. On the contrary, if Alice is associated with her consciousness, she has no free will. Consider, for instance, the following sentence: “I can have true free will: I can have true alternatives, true freedom to choose among them, true will to cause what I have decided, and eventually true responsibility” (Tononi et al., 2022). For CET, the validity of this statement depends on how the “I” is conceptualized.

This also implies that not integrated information of irreducible causal mechanisms (Tononi, 2008; Oizumi et al., 2014), architecture peculiarities of neural networks (Dehaene and Naccache, 2001; Baars et al., 2013), cognitive processing (Clark, 2013), or higher-order linguistic thoughts (Lau and Rosenthal, 2011; Rolls, 2012) but free volition, inherited by the brain from fast and random reflexes rooted in chemo-, magneto-, and photoreceptor cells which are very sensitive to *quantum* effects (Arndt et al., 2009; Brookes, 2017; McFadden and Al-Khalili, 2018) is the main obstacle that prevents computer scientists from making deterministic machines conscious.

## The Stream of Consciousness in Brain Dynamics

Consciousness has been a prescientific concept with a number of different connotations that relate to various aspects of conscious experience. The science of consciousness must rely on the fact that consciousness is a dynamic process, not a thing or a capacity (James, 1950). It should also take into account that the observation of single cells activity has little to say about mental processes. Large-scale interactions between neural networks are therefore more important than the contribution of individual neurons *per se*. Thus, only neural dynamics at the macro-scale can account for global brain states accompanied by conscious experience. Most attempts to understand the neural mechanisms of consciousness have proceeded by searching for the “neural correlates of consciousness” or NCC (Crick and Koch, 2003). However, correlations by themselves do not provide explanations and there is a need for a framework connecting fundamental aspects of conscious experience at the phenomenal (psyche) level to the corresponding aspects of brain dynamics at the underlying causal (hard) level.

The evolution of consciousness depends on the physical *continuous* dynamics of the brain, comprising about 10^11^ neurons connected to each other by thousands of synapses. Clearly, consciousness not only requires neural correlates with their appropriate anatomical architecture but also the time to be processed. A wealth of experimental evidence suggests that conscious experience is a discrete phenomenon processed unconsciously at the soft level (see Section “Temporal resolution of the stream of consciousness”). Moreover, a pure biophysical approach to studying neural activity at micro- and mesoscopic scales cannot account for subjective, internally generated mental phenomena without resorting to their contextual emergence at the macroscopic scale (Atmanspacher and beim Graben, 2007). It has been pointed out many times that there should be an optimal spatio-temporal grain size at which the brain can generate phenomenal experience (Tononi, 2008; Chang et al., 2020). In general, the correspondence between conscious states and neural processes should be provided by mapping brain dynamics onto the state space of consciousness.

CET compresses all these requirements into three key prerequisites:

1. **Physicalism** (mind-brain identity): consciousness depends entirely on brain activity governed by natural laws at the hard level, not on anything else;
2. **Dynamism** (temporality): consciousness not only requires the NCC but also the time to be cognitively processed at the soft level;
3. **Contextuality** (scale-dependence): only large-scale brain dynamics can account for the emergence of conscious states at the psyche level.

A principled distinction between CET and classical theories is that CET involves quantum indeterminism. On the other hand, while quantum-inspired theories engage quantum computing across the whole cortex to account for consciousness and free volition, CET makes minimal use of quantum randomness placed into the arousal nuclei of the brainstem to initiate cognitive processing due to stochastic brain dynamics that are classical in nature. Thus, CET is a semi-classical physical theory of consciousness.

### Deriving consciousness from brain dynamics

Based on the three prerequisites, CET will model consciousness as the stream of macrostates, each specified by a particular structural-functional configuration of the whole-brain network *𝒩*, where NCC ⊆ *𝒩*. Here 𝒩 stands for a graph *G* = (*N*, *E*), where *N* = |𝒩| is the set of nodes (ideally, neurons), and *E* ⊆ *N × N* is the set of edges (ideally, synapses). Because it is computationally impossible to operate on *N* ≈ 10^11^, the first step in formalizing the stream of consciousness is to approximate large-scale brain dynamics at the hard level. To derive the stream from the SOC approach, CET refers to the Langevin formalism as a most general description of a system that depends upon a deterministic flow *y* and stochastic fluctuations *ω* [which, note, do not discriminate between quantum (i.e., ontic) and statistical (i.e., epistemic) randomness, e.g., in Brownian motion]


(2) dψ=−γψ(t)dt+dω(t)

The formalism can then be transformed into different models depending on the way researchers will adopt that formalism in their study. Those models may be biophysical (mean-field) approximations (Breakspear, 2017; Parr et al., 2020) or phenomenological (synchronization) models such as Stuart-Landau, Kuramoto, Haken-Kelso-Bunz, and other models (Cofré et al., 2020; Kelso, 2021; Kraikivski, 2022; Vohryzek et al., 2022).

Here *ψ* (𝒩, *t*) is a descriptive function whose representation by the order parameter in a phase space *O* should account for metastability, avalanches, and SOC of the global neural activity wandering among dynamical attractors (Kelso, 1995). Originally grounded in physics, chemistry, and biology (Bak, 1996; Jensen, 1998; Haken, 2004), SOC is thought to be of crucial importance in neural activity as it poises the brain on the edge between order and disorder near a bifurcation (Blanchard et al., 2000; Chialvo, 2010; Beggs and Timme, 2012; Deco et al., 2015). This allows the neural network 𝒩 to exhibit the properties of scale-free networks and to produce flexible patterns of coordination dynamics at the hard level. This, in turn, can generate a repertoire of different conscious states at the psyche level, thereby increasing an adaptive behavioral response of the organism to given environmental stimuli (Hesse and Gross, 2014; Cocchi et al., 2017; Dahmen et al., 2019).

CET *postulates* the emergence of consciousness from brain dynamics at critical points as its derivative extracted in discretized time *τ*,


(3) $$S(\tau )\overset{\text{def}}{=}\frac{d\psi }{d\tau }$$

The continuous/discrete dichotomy appears naturally between the brain dynamics, described at the causal (hard) level, and the stream *S*(*τ*) of conscious states, presented at the phenomenal (psyche) level. In effect, Equation (3) should capture the instantaneous transitions from continuous brain dynamics to discretized conscious states, identified with a single point *o* ∈ *O* in a phase space. The phase transitions in brain dynamics occurring at discrete moments of time can then be viewed as the manifestation of pulsating consciousness in the framework of cinematic theory of cognition (Freeman, 2006). This approach finds now experimental evidence in many studies (Haimovici et al., 2013; Mediano et al., 2016; Tagliazucchi, 2017; Demertzi et al., 2019; Kim and Lee, 2019) showing that only states integrated near criticality can ignite conscious experience in the brain.

CET suggests a simple mathematical analogy between consciousness and the physical force, derived from the momentum in Newtonian mechanics,


(4) F=dp/dt=ma

Seeing consciousness as a “mental force” seems to be more moderate and accurate, at least ontologically, than viewing consciousness as a fundamental property of matter like mass, charge, and energy (Tononi, 2008). The analogy between conscious experience and mass, advocated by IIT, is based on a quantitative account of the level of consciousness measured by integrated information Φ a single complex of irreducible causal mechanisms might generate (Oizumi et al., 2014). IIT argues: if a complex can generate Φ, no matter whether it is organic or not, it will have consciousness (Tononi and Koch, 2015). Instead, CET brings into focus the dynamism of consciousness. The analogy with force goes in line with the fact there is a tiny but principled distinction between mass and force in physics by Equation (4): the former is a scalar quantity which is indeed constantly intrinsic to a system, whereas the latter is a dynamical characteristic of motion defined by a vector quantity of a system’s action that can vanish in inertial states.

Similarly, Equation (3) represents consciousness as a dynamical characteristic of the neural network 𝒩 not as an intrinsic potency of causal mechanisms in the brain or anywhere else. According to this conceptualization, the brain has no mental force if its dynamics depart from criticality, as it occurs in unconscious states such as coma, sleep, or general anesthesia (Hudetz et al., 2014; Tagliazucchi et al., 2016; Golkowski et al., 2019; Huang et al., 2020), but not in resting states where neural activity preserves criticality (Deco and Jirsa, 2012; Barttfeld et al., 2015). On the other hand, even in critical dynamics, the brain lacks the mental force during some interval Δ*t* until a next conscious state is unconsciously processed.

**Figure 1:**
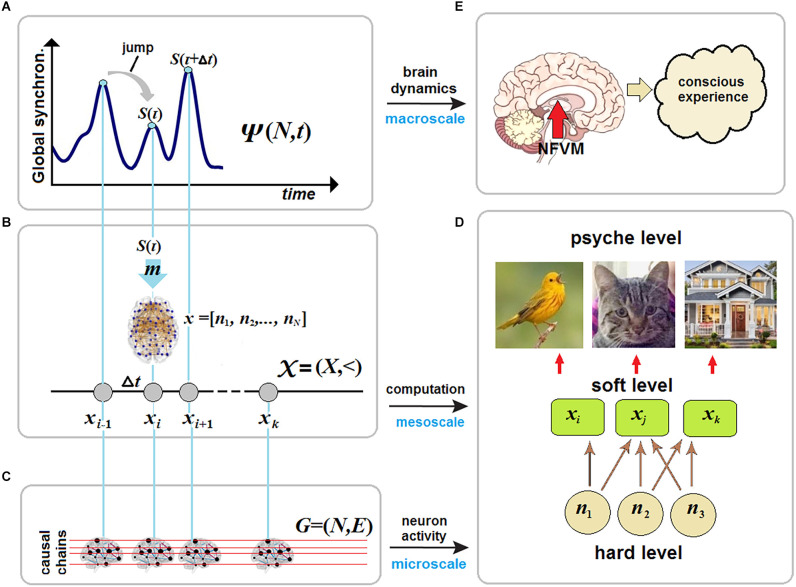
Brain criticality and conscious experience. **(A)** In large-scale brain dynamics, conscious states emerge at critical points near phase transitions between synchronization (order) and desynchronization (disorder) patterns of neural activity at the microscale. **(B)** The map *m* transforms each critical point of brain dynamics, described in a phase space, onto the whole-brain network 𝒩 in a vector space as a particular NCC responsible for a certain conscious state at that moment of time. **(C)** The stream of consciousness can then be formalized as a discrete chain of states (*N*-dimensional vectors) and studied in causal dynamical modeling as a directed acyclic graph. **(D)** In neural pleiotropy, many neurons constitute a particular NCC for producing a certain conscious percept, thereby involving a single neuron in generating very different percepts. **(E)** Conscious experience is a product of unconscious computations initiated by the brainstem at the hard level and accomplished by various thalamocortical systems at the soft level.

To make the above analogy more comprehensive, imagine a clockwork toy, say, a jumping frog. The engine of the toy will impel it to iterate the same jump over and over. The force occurs only at discrete moments of jump-initiation between which motion decays. In critical dynamics, the brain exhibits flexible patterns of coordinated dynamics which decay after some critical value (Gollo et al., 2012; Tognoli and Kelso, 2014). Conscious experience can be ignited only on the edge between the two phases as if the brain accumulated information for triggering the next “jump” with some mental force at discrete moments of time (Figure 1A).

### Temporal resolution of the stream of consciousness

According to Equation (3), the brain needs some time to process a new conscious state from the previous one. There are two complementary ways to estimate the interval Δ*t*– either by monitoring brain dynamics to calculate phase transitions at the hard level or by obtaining a subjective report at the psyche level. Unfortunately, both approaches do not provide an exact estimate. The monitoring of brain dynamics is non-trivial because of the heterogeneous intrinsic timescales involved. An averaged interval is usually reported to be about 200 ms (Kozma and Freeman, 2017; Deco et al., 2019). The second approach, based on first-person reportability, is affected by the problem of multiple temporal resolutions (White, 2018).

To comprise both approaches, we assign the interval to a wide window Δt ≈ 100–450 ms that encompasses multiple experimental findings—from the periodicity of attentional cycles at approximately 7–13 Hz (VanRullen et al., 2014) to the attentional blink on masked targets separated by 200–450 ms (Shapiro et al., 1997; Drissi-Daoudi et al., 2019). Yet, an important neurophysiological aspect of brain dynamics is that the stream *S*(*τ*) cannot be normally delayed for a period longer than about 300 ms according to the timescale proposed to be crucial for the emergence of consciousness (Dehaene and Changeux, 2011). Consciousness spontaneously fades after that period, for example, in anesthetized states (Hudetz et al., 2014; Tagliazucchi et al., 2016).

Only states that emerge globally and are integrated at critical points are conscious. In the stream, each state appears instantaneously as a snapshot accompanied by a phenomenal percept of the “specious present” (Varela, 1999), which provides human (and most likely animal) observers of the external world with an egocentric frame of reference, preserving its stability over time (Seth and Baars, 2005). Due to the cognitive updating of this self-referential frame by the acquisition of new contents, consciousness remains well informed at the psyche level about what is occurring around. Thus, while conscious states emerge only at discrete critical points of brain dynamics, subjects can still feel the continuity of being and acquire the illusion of a persistent presence.

There are also arguments for a continuous flow of conscious experience (Fekete et al., 2018; Kent and Wittmann, 2021). However, these are typically grounded in the phenomenology of consciousness and time instead of being based on the neuroscience of consciousness and the physics of time. Yet, they do it without making a difference between the rigorous notion of mathematical continuum and something that can be continued. If such arguments have any merit, their core can be formulated like this: “If conscious experience is produced by the brain, then it would seem that there must be a lawful relation between the state of the brain at the given time and the percept that the person is experiencing… because experiencing or perceiving is an activity, not something to be looked at” (Brette, 2019). Indeed, such a “lawful relation” must be suggested.

Equation (3) explains how *discrete* conscious snapshots at the psyche level can be separated from *continuous* brain dynamics at the hard level. Moreover, because consciousness does not observe how the experience was processed by the brain, awareness requires no time to be ignited. Since the ignition across the brain’s workspace occurs phenomenally due to SOC, there is no dynamical process that should transmit information into a special site of the brain to reach the subject’s awareness. Experience is the information the brain has unconsciously computed at that specious moment τ. The illusion of temporal continuity of consciousness is merely a trivial corollary of its self-evidential nature: consciousness cannot in principle detect its own absence in the brain. Whenever consciousness searches for itself, the search happens successfully for an obvious reason: in the stream, consciousness will always be present in introspection. Likewise, whenever we look in a mirror, we always see ourselves as if we were constantly there. We know that it is not true, but it is impossible to catch the mirror before our image will appear.

In particular, the discreteness of conscious experience explains how the subjective distortion of perceived temporal duration produced by emotionally charged events and causing the sense of dilated time in the stream *S*(*τ*) can occur. Overestimation of time is associated with many factors such as enhanced memory encoding or intensive involvement of the limbic system and the interconnection with medial cortical areas (Dirnberger et al., 2012). This depends on how the states were unconsciously processed over multiple timescales in brain dynamics (Merchant et al., 2013) while their sensory-cognitive contents had been compressed over temporal chunks *T* = ∑ Δ*t*, composed of non-overlapping intervals. Otherwise, for the sake of continuous conscious perception, we should agree on something like space-time effects in Relativity theory as if a perceived chunk *T*, as compared to a physically real *T*^*R*^, would indeed be dilated by the Lorenz transformations $T=T^{R}/\sqrt{1-\upsilon^{2}/c^{2}}$. Thus, the discrete model of the stream *S*(*τ*) proposes a more reasonable and parsimonious explanation of the subjective distortion in time perception as merely depending on a ratio *T*Δ*t*, i.e., on a variation of Δ*t* during which those emotionally charged conscious states were processed.

The passage of time (whatever it is physically) cannot be perceived at all without sensory-cognitive contents processed by different brain systems at the soft level and integrated globally at the psyche level. This also explains why time perception vanishes completely in altered states of consciousness such as sleep, anesthesia, or epileptic seizures. It occurs as brain dynamics deviate from criticality upon which discrete conscious experience entirely reposes (Tagliazucchi et al., 2016).

### The stream of consciousness as a causal chain of discrete states

Now let *m*: *O* → *V* be a map from the phase space *O* onto a vector space *V* over the product *N* × *N* of all neurons of the brain network 𝒩. The map returns *S*(*τ*) from a point *o* ∈ *O* to an *N*-dimensional vector *x* = [*n*_1_, *n*_2_,‥., *n*_N_], where *n*_i_ = 1 or *n*_i_ = 0 stand for neurons active or inactive at the given time. We write (omitting details),


(5) $$S(\tau )\overset{m}{\to }x$$

Thus, each state *S*(*τ*) is represented now by *x* as a certain structural-functional configuration of 𝒩 that is responsible for that subjective snapshot at the moment τ. The discreteness of the stream signifies that all the conscious states could, at least in principle, be naturally enumerated from a subject’s birth, not merely by a lag in experimental settings.

Let the brain bring consciousness to a state *x*_i_ at the moment *τ* = *t*. We can rewrite Equation (5) as follows.


(6) $$\psi \;(𝒩,t)=x_{i}$$

The next conscious state will emerge over the time interval as


(7) $$\psi \;(𝒩,t+\Delta t)=x_{i+1}$$

In the timeless description, the stream *S*(*τ*) is a discrete causal chain *𝒳* = (*X*,<) (Figure 1B), where *x_i_* ∈ *X* and whose relation < standing for the causal (and temporal) order is transitive and irreflexive. Here the irreflexivity means ∀ixi<xi that forbids closed causal loops and, in particular, instantaneous feedback circuitry in brain dynamics that might somehow enable consciousness with causal power over the brain, for example, due to the presumed quantum temporal back-referral of information in Orch OR (Hameroff and Penrose, 2014). CET strictly rejects the possibility that consciousness could—classically or quantum-mechanically—choose a state to arrive at.

Consciousness is a physically classical macro-phenomenon (though quantum-triggered) that is always local in space and time. For consciousness, planning something, as discussed in Alice’s scenario, does not mean to be already in that state. In other words, every state in *S*(*τ*) is actual, and any chosen state should also be actual. However, it is physically impossible for the brain or for the stream of consciousness to be ahead of itself. Physically, the brain is exactly where it is. Mentally, consciousness is what it is just now. Even assuming quantum temporal back-referral, closed causal loops would be involved, as if consciousness would be now in a state it had already chosen in the state before. On any account, this would imply that before choosing a state consciousness should already be in that state despite irreflexivity.

Herzog et al. (2016) suggest a discrete model of consciousness very similar to that of CET. The authors assume that conscious states represent integrated, meaningful outputs of unconscious (modular) computations carried out at the soft level which had been causally provided by dynamical attractors at the hard level of interacting neurons. They compare a conscious snapshot with a *k*-dimensional feature vector *V_i_* that contains a value for each relevant feature *i* of an external stimulus (e.g., color, size, shape, or position), which together constitute a meaningful post-hoc representation of the event processed unconsciously during an interval Δ*t*. The interval is the period of sense-making (Herzog et al., 2016). CET transforms their model, based on a statistical presentation of an external stimulus by a feature vector *V_i_*, into an internal neuron-based representation of that stimulus in the brain network 𝒩. Namely, in the chain *𝒳* = (*X*,<), each snapshot *S*(*τ*) is a particular NCC described by a *N*-dimensional vector *x_i_* = [*n*_1_, *n*_2_,‥., *n*_N_] that constitutes a given conscious state which is responsible for the perceived stimulus at that time.

The stream of consciousness can now be represented in terms of dynamical causal networks formalized typically as a directed acyclic graph *G*_*u*_ = (*N,E*), where edges *E* indicate not synaptic connections but cause-effect pairs among a set of nodes *N* which in turn represent a set of associated random variables with a state space $\Omega =\Pi_{i}\Omega_{N_{i}}$, based on a given set of background conditions (states of exogenous variables) *U* = *u* (Albantakis et al., 2019). The representation can be made by mapping the temporal evolution of the brain network 𝒩 onto the acyclic graph. Accordingly, the chain *𝒳* can be defined in *G_u_* by a partition of its nodes *N* into temporally ordered slices, *N* = *N*_1_, ‥., *N*_*k*_, each interpreted as a particular NCC of a discrete dynamical system starting with an initial slice *N*_1_ ≅ *x*_1_ and such that the parents of each next slice are fully contained within the previous slice. This definition prohibits any instantaneous causal loops and signifies that *G_u_* (hence *𝒳*) fulfills the Markov property (Figure 1C).

## Neural Correlates of Consciousness

According to Equation (5) each conscious state *S*(*τ*) in brain dynamics can be represented by *x_i_* as a particular NCC. The NCC has been traditionally defined as the minimal set of neuronal events that gives rise to a specific aspect of a conscious percept. In the context of the binding problem, the NCC should then be seen not as the static connectivity patterns over anatomical brain regions but as dynamical and transient networks with synchronous neural activity as those evolve across the whole brain in critical dynamics. Klein et al. (2020) argue for what can be called “neural pleiotropy” in the search for NCC. In biology, pleiotropy refers to the fact that a single gene can affect a number of otherwise distinct phenotypic variables.

Accordingly, it can be said that not only many neurons from different brain areas simultaneously contribute to a single conscious state *x_i_* (a particular NCC) at that moment *τ* but also many conscious states are affected by a single neuron, e.g., *via* selective participation in producing neuronal avalanches (Bellay et al., 2021). Thus, a conscious experience at the psyche level involves multiple phenomenal elements, many of which can occur in the context of other experiences (Figure 1D). Thus, one might then consider how different NCC might be mapped onto a state space of phenomenal experience to account for qualia at the psyche level (Kraikivski, 2020). However, CET specifies that the problem of the privacy of consciousness cannot be solved abstractly. Secured privacy is guaranteed by the NFVM, preventing the possibility of copying one’s unique consciousness.

Generally, the search for a physical substrate S for some P makes sense if P is a well-defined phenomenon accessible to objective experience. Here, the *objective* can be replaced by the *collectively subjective*, something that everyone can experience independently. For example, if P means the Moon, this is well-defined under objective experience: we all understand that the Moon means an object in the night sky which is accessible for our public evidence. Further, physicists tell us that the physical substrate S of this P consists of atoms that are already inaccessible to our public evidence. Instead, we simply believe physicists, and, in principle, everyone can verify their belief in a lab. Yet, some people may also believe in ghosts revealed to them in their subjective experience, but, unlike atoms, these might not even in principle be tested in a lab for public evidence.

Likewise, when one speaks of consciousness, we all understand what it means. But this produces a lot of confusion. Consciousness is ontologically like neither one of these. Unlike the Moon, consciousness cannot be in principle the object of our collective experience. Unlike an atom, we do not even need to believe in its existence because of its self-evidential nature. Unlike a ghost, it is naturally revealed to all of us with no need to verify its presence in a lab. Although consciousness is the necessary prerequisite for the public evidence of the existence of anything, it itself is neither well-defined nor even a phenomenon under objective experience. Thus, the empirical search for NCC cannot be theory-neutral but depends on how consciousness is conceptualized (Pauen, 2021). How might the NCC be detected without knowing what exactly the function of consciousness is and how it has evolved?^2^

The NCC program, as initiated by Crick and Koch, has been explicitly based on the idea of active consciousness. This allowed the authors to propose the search for neural correlates of visual consciousness which should “produce the best current interpretation of the visual scene in the light of past experience …and to make this interpretation directly available, for a sufficient time, to the parts of the brain that contemplate and plan voluntary motor output” (Crick and Koch, 2003). The program had quickly been divided into two parts: the search for the level of consciousness and the search for the cognitive (reportable) contents of specific NCC (Miller, 2007; Hohwy, 2009). The former was explicitly concentrated on the diagnostic assessments of coma/level of arousal in humans, tested for instance with the Glasgow Coma scale or Coma Recovery Scale-revised, while the latter remained concerned with conscious (mostly visual) perception vs. unconscious processing in experimental studies on awake subjects.

These in turn generated a lot of cognate concepts such as prerequisites of consciousness (preNCC), proper correlates (NCCpr), their consequences (NCCcons), and others (Bachmann and Hudetz, 2014; Northoff and Zilio, 2022). CET is not involved in all of these. CET denies active consciousness, thereby reducing the importance of cortical regions for the study of NCC. CET argues that the search for true NCC suggested as an “easy part” of the hard problem (Crick and Koch, 2003) is an attempt to sidestep the privacy of consciousness in the same way as the Turing test attempts to do it by obtaining a machine report.

Instead, CET decomposes the concept of NCC into separated neural configurations that are responsible for different conscious states. In principle, one can uncover an NCC for any particular state *x* = [*n*_1_, *n*_2_,‥., *n*_N_] by merely detecting activity patterns in 𝒩 at that moment τ. The minimal neural substrate can then be defined by the intersection of all those states over the stream *S*(*τ*), or, more generally, as


(8) $${\text{NCC}}^{\min}=\cap_{i=1}^{2^{N}}x_{i}$$

Here 2^*N*^ is a set of all possible states (from full vigilance to sleep, or coma) a subject might have during his lifetime. To identify which minimal correlates are necessary for consciousness, we need to associate it with the most primitive core of subcortical consciousness presented in infants born without the telencephalon (Damasio et al., 2012; Aleman and Merker, 2014; Barron and Klein, 2016; Panksepp et al., 2017) or even in patients with the unresponsive wakefulness syndrome (see Section “Mental force in critical brain dynamics”).

Otherwise, if we assume an active role of consciousness (i.e., free will) in an attentional effort, active inference, decision-making, planning, and goal-directed behavior, the NCC would comprise most of the brain, including even the cerebellum (Schmahmann, 2010; Friston and Herreros, 2016) that is generally not considered in the NCC debate,


(9) $${\text{NCC}}^{\text{active}}=\cup_{i=1}^{2^{N}}x_{i}$$

The general idea of linking consciousness to volition and cognition relies on the observation that they seem always to be associated with each other (Bachmann and Hudetz, 2014; Naccache, 2018; Aru et al., 2019). However, the premise that consciousness is active in brain dynamics claims much more than the NCC program has suggested—to account for the minimal neural substrate that is necessary and sufficient to ignite consciousness in critical points of brain dynamics. The premise makes legitimate the use of such terms as “conscious processing” in parallel with unconscious processing.

CET denies the possibility of conscious processing that would be active and continuous over the stream *S*(*τ*) of discrete conscious states. This argues that the integration of neural activity, associated with a particular conscious state emerging at the psyche level, cannot be dissociated from underlying cognitive and volitional processes at the soft and hard levels respectively. Some regions in the front or in the back of the cortex that are thought to be relevant in the NCC debate (Boly et al., 2017) are also involved in various functions that are necessary for volition and cognition, e.g., the supplementary motor area in Libet-type free will experiments, or the inferior parietal cortex and many corticothalamic loops in predictive processing. However, in CET, consciousness is derived from brain dynamics only as a mental force, it cannot persist without volition and cognition, and completely fades if there is no space for the cognitive evolution when brain dynamics depart from criticality (as in coma, epileptic seizures, and general anesthesia).

Thus, contrary to the idea of active consciousness, advocated by many theories of consciousness, CET takes an “inverted perspective”: consciousness is a passive phenomenon, ignited at critical points of brain dynamics and resulting from unconscious computational processes implemented by various thalamocortical functional systems. When distilled from those processes, conscious states emerge as a mental marker of brain dynamics, by analogy with the physical force as a dynamical marker of a moving system. This is the subjective experience that makes a difference over time from its own perspective, thereby acquiring the illusion of causal power over neural activity^3^.

Instead of discussing different brain regions with their contributions to subjective experience, e.g., the prefrontal cortex vs. posterior “hot zones” (Koch et al., 2016; Boly et al., 2017), or dissociating attention from consciousness (Koch and Tsuchiya, 2007; Nani et al., 2019), CET argues: conscious states emerge as neural configurations *x* = [*n*_1_,‥.,*n*_N_] at critical points of brain dynamics, represented then as different NCC. Consciousness has not played any special role to have its own evolution-driven neural substrate; it emerges passively from volitional-cognitive substrates distributed across all regions of the brain (and from primitive organisms to humans). There is no special NCC that might causally influence brain dynamics, NCC^active^ = ∅. Instead, the NFVM initiates a random micro-event which can be stochastically amplified by bottom-up causation *via* neuronal avalanches to generate a particular conscious state. Such avalanches are intrinsic to and ubiquitous in critical dynamics (Beggs and Plenz, 2003; Hahn et al., 2010; Shew et al., 2011).

## Volition in The Stream of Consciousness

To account for quantized neuronal events that might cause avalanches across the brain, CET places the NFVM into the brainstem (Figure 1E). This derives from the fact that the brainstem is responsible for spontaneous arousal and vigilance conducted through the ARAS projecting to the thalamocortical systems (Paus, 2000; Merker, 2007). Although the cortex is mostly responsible for elaborating conscious contents, damage to ARAS and intralaminar nuclei of the thalamus can abolish consciousness (Mhuircheartaigh et al., 2010; Edlow et al., 2020). Moreover, the neuromodulatory influences of the brainstem (due to its anatomical location in the neural hierarchy) act as control parameters of SOC, moving the whole cortex through a broad range of metastable states, responsible for cognitive processing in brain dynamics (Bressler and Kelso, 2001). The NFVM is thus a natural trigger of critical dynamics over which the stream of consciousness evolves.

Placing the NFVM into the brainstem is also related to the fact that the brainstem is the oldest brain region that plays a fundamental role in the maintenance of homeostasis (Parvizi and Damasio, 2001). Evolutionarily, brains have evolved gradually as multilevel systems consisting of anatomical parts that were selection-driven as adaptive modules for executing specific functions. Any brain function requires an appropriate neuronal structure able to generate various dynamical patterns to carry out that function optimally. It is well-known that the global architecture of the brain is not uniformly designed across its anatomical parts, which structural signatures are specialized under corresponding functions. Possibly, the network characteristics of the brainstem with its reticular formation were developed to be especially conducive to small neuronal fluctuations. These small neuronal fluctuations were then amplified to account for reflexes and primary volitional reactions. Later they were projected to higher thalamocortical systems to make computations at the soft level.

Thus, CET reduces the active role of consciousness to the brainstem activation of each conscious state, triggered by the NFVM [this would happen in the same way as, for instance, long-lived quantum entanglements in the cryptochromes of the retina are thought to participate in precise magnetoreception of the avian compass (Ritz, 2011; Hiscock et al., 2016)]. The NFVM is necessary to warrant the indeterminism of the stream based on quantum randomness as emphasized above (Conway and Kochen, 2008), not merely on a stochastic (deterministic) account of brain dynamics. Thus, in the stream *S*(*τ*), all conscious states (but not consciousness) must be initially Bell-certified against hidden deterministic variables *λ* by Equation (1) to be independent from the past.

Many researchers commonly agree that conscious states represent a simultaneous binding of neural modules, but there is no agreement regarding the NCC underlying this form of integration. CET addresses the problem by dividing the NCC into two parts: a first one that initiated a new conscious state, and a second part that integrated the cognitive contents of that state. The neural correlates (NC) of the NFVM (located in the brainstem) provide the neural basis for the integration of all states processed by the thalamocortical systems. This makes the NFVM a necessary and sufficient mechanism to maintain the sleep-wake cycle in patients with unresponsive wakefulness syndrome (UWS) after widespread thalamocortical damage when brainstem activity is more or less preserved (Laureys et al., 2004),


(10) $${\text{NCC}}^{\min}=\text{NC (NFVM)}\overset{\text{def}}{\text{=}}\text{UWS}$$

Recovery of consciousness depends then upon the functional reemergence of the ARAS, which must provide sufficient input *via* the thalamic projections to the anterior forebrain mesocircuit and frontoparietal network (Edlow et al., 2020). Indeed, it is known that full recovery from UWS can be accompanied by restoration of activity in frontoparietal areas (Giacino et al., 2014). In contrast, brainstem lesions cause immediate coma by damaging the ARAS and its associated neuromodulatory systems (Parvizi and Damasio, 2001). Thus, if the NFVM is severely damaged, no conscious state could be initiated in brain dynamics, even when thalamocortical systems remained completely or partially intact,


(11) $$\text{NC (NFVM) = }\emptyset \overset{\text{def}}{\text{=}}\text{coma}$$

The NFVM could also account for subcortical conscious experience in infants born without the cortex, on the condition of some preserved islands of the limbic system coupled with arousal centers. This goes in line with Merker’s proposal that the stream of consciousness derives from the interactions within the brainstem, supporting the triangle of action-target-motivation, while the thalamocortical systems provide cognitive contents for “immediate, unreflective experience” (Merker, 2007). For example, experimental evidence shows that decorticated rats preserve consciousness driven by volition (NFVM) from the brainstem, but often exhibit hyperactive wandering and more emotional behavior than their neurologically intact siblings whose behaviors are just suppressed and cognitively modulated by the cortex (Panksepp et al., 1994).

## Consciousness as A Mental Force

In Newtonian mechanics, the force is a time derivative of the momentum. But there are no things such as the force and the momentum; these are only the dynamical characteristics of a moving system. Similarly, consciousness can be derived—in any meaningful sense—only from the cognitive evolution of the brain over lifetime. Consciousness is thus no more real than the force in physics though the latter can be measured and calculated. Hence, if we will proceed with this conceptualization of consciousness as a mental force, we need to propose objective observables that could measure its magnitude at a given time.

### Mental force in critical brain dynamics

Before suggesting a candidate measure for consciousness let us return to the metaphor with a clockwork frog. The only operation a frog can execute is a jump initiated by its engine repeatedly with the same magnitude of physical force, *F* = *ma*. The frog will make the same jump over and over regardless of how the environment changes. To continue the analogy between this force, triggered by the frog’s engine at discrete moments of time, and the brain’s mental force, ignited momentarily at critical points of brain dynamics, we need to compare the iteration of the same jump with the alternation of conscious states.

In the stream, each state must make a *difference* from the past (Edelman, 2003) to gain an insight into what occurs around it. Thus, a mental jump at the psyche level over discretized time *τ* provides consciousness with new knowledge that can be viewed as Bayesian updating of prior beliefs in the framework of predictive processing models at the soft (unconscious) level. What follows is that the same conscious state, experienced by a subject repeatedly, would be like a freeze-frame on a TV screen, yet, preserving the same mental force. The brain, generating every time the same snapshot, could not make a difference from the past to learn something. Accordingly, memory networks should have nothing to update over the stream of such states.

Perhaps, just these conditions are presented in UWS that, unlike coma, is accompanied by spontaneous eye and limb movements without evidence of goal-oriented behavior or sensory responsiveness (Giacino et al., 2014; Schiff et al., 2014). According to Equation (10), in these conditions, the NFVM should still be preserved within the brainstem to maintain arousal cycles with the lowest level of conscious experience, whereas thalamocortical systems responsible for cognitive (unconscious) processing would be severely suppressed. It also explains why patients who recovered after UWS have no memories of their staying in that state as if time perception was also broken (Gosseries et al., 2014).

Now replace the frog with a Bell-certified random number generator producing a string of numbers a Turing machine could not compute. Let the numbers symbolize conscious states, totally disconnected over the stream. The stream of such states would be unpredictable in favor of free volition. On the other hand, as the brain has to make its own predictions about the world, cognition and logical reasoning should be unguided in this case because a subject having those states could not concentrate on a task to gain a coherent insight into it. The subject could then be viewed as going among many arbitrary frames (thoughts), while the information gain (as estimated by the Kullback-Leibler divergence between the prior and the posterior), would be too big to make a consistent difference from the past.

Perhaps, similar conditions might characterize attention deficit hyperactivity disorder (ADHD) in the literature. Its typical symptoms are disorganization, impulsiveness, trouble with focussing on tasks, and low attention span (Salmi et al., 2020). In this disorder, consciousness, as the *way of being* (Tononi, 2008), is ill-adaptive to the environment. How can free volition be manifested there? CET predicts that ADHD can occur when the random neural event initiated by the NFVM and transmitted by the ARAS up to the thalamocortical systems cannot be normally modulated by these parts of the NCC. Whatever the neurophysiological reasons of it may be, there is evidence that critical phenomena are involved in ADHD (e.g., Zimmern, 2020; Heiney et al., 2021; Rocha et al., 2022).

### Neural complexity as a measure of consciousness

**Figure 2:**
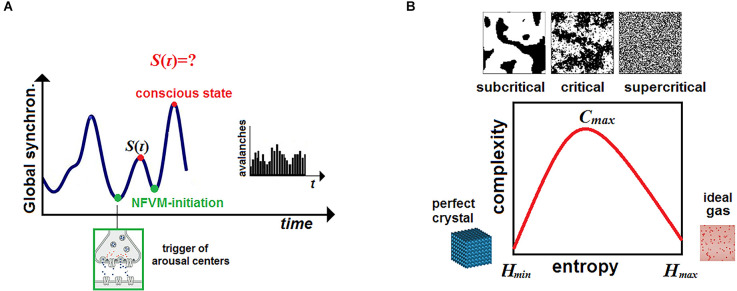
Critical dynamics, phase transitions and the NFVM. **(A)** In critical dynamics, one neuron excites another and causes avalanches of activity spreading across 𝒩 and obeying the power law of distribution. The brain exhibits a broad range of flexible patterns of coordinated dynamics which decay after some critical value. While the NFVM initiates avalanches from arousal centers, a decentralized feedback mechanism arises spontaneously to lead a self-organized system from subcritical to supercritical phases. **(B)** A simulation of critical phase transitions on the 2D Ising spin lattice model exhibits different degrees of synchronization or coordination (black dots) from subcritical to supercritical states as temperature (a control parameter) increases from left to right. Adopted from Kitzbichler et al. (2009). Complexity as an entropy-based measure placed between two thermodynamic extrema: a perfect crystal at absolute zero temperature and an ideal gas. *C*_𝒩_ reflects a mixture of integration/segregation in brain dynamics with maximal values near criticality between subcritical and supercritical phases presented above.

According to Equation (3), conscious states can emerge only near criticality while the brain moves from a subcritical regime where excitation dies out to a supercritical regime accompanied by neuronal avalanches (Haldeman and Beggs, 2005; Chialvo, 2010). SOC maintains a long-term correlation of brain regions to provide optimal information storage and computational power on the edge between order and disorder (Figure 2A). In CET, consciousness is like a river buoy fluctuating on the surface of water regardless of its depth. The behavior of such a float can well characterize underwater dynamical processes (for example, in fishing or navigation) without, however, having any influence on those complex and invisible processes.

Similarly, conscious experience is what has been just exposed to the psyche level by the brain at a given time; it is a passive marker of the deep neural processes going at hard and soft levels of brain dynamics. Now we can suggest a candidate measure to estimate the mental force of this fluctuating phenomenon, generally referred to as the level or quantity of consciousness. Recently, SOC has been proposed as a determinant for complexity measures of consciousness, mainly with respect to the concept of integrated information in IIT, based on the fact that both statistically reflect the same interplay between integration and segregation in network dynamics exhibiting scale-free patterning (Sporns, 2006; Lee et al., 2010; Tagliazucchi, 2017; Aguilera, 2019; Kim and Lee, 2019). While the critical dynamics are characterized by the order parameter, for example, a mean proportion of activated neurons in 𝒩, with the control parameter depending on connectivity density over time (Hesse and Gross, 2014), the complexity measures evaluate the degree of integration of 𝒩 in a particular state (Timme et al., 2016; Rosas et al., 2018).

In CET, the consciousness’ objective observables, derived from brain dynamics at discrete moments τ, will be the neural complexity measure *C*_𝒩_ (Tononi et al., 1994). This would be a more feasible measure, instead of its sophisticated version (integrated information Φ) that requires computing distance measures between probability distributions of a system and finding the minimum information partition (Oizumi et al., 2014). In IIT, Φ is determined by a major complex of irreducible causal mechanisms, which is then identified with NCC. The emphasis on the *ontological* status of Φ takes some form of panpsychism, and includes the possibility to ignite consciousness in deterministic machines (Tononi and Koch, 2015). Unlike IIT, in which the presence of consciousness itself is conditioned on integrated information a system is able to generate, CET determines the stream of consciousness by SOC in Equation (3). The complexity measure *C*_𝒩_ has no fundamental property. It is needed only as an objective (*epistemic*) observable to evaluate the mental force of brain dynamics at the given time without assuming machine consciousness or its ubiquity in matter like mass.

The main reason to accept *C*_𝒩_ as a quantitative measure of consciousness is that the notion of complexity is commonly conditioned on the same balance between order and disorder. In statistical physics, it starts by considering the perfect crystal and the isolated ideal gas as simple classical models of two extrema. The former represents a maximally ordered state of a system with entropy *H* = 0, and the latter symbolizes its maximally disordered state with *H* = *H_max_*, while both have zero complexity (Lòpez-Ruiz et al., 1995). Yet, just like the emergence of consciousness conditioned on large-scale brain dynamics with characteristic temporal scale Δt ≈ 100–450 ms in CET, the complexity is a scale-dependent concept. This cannot be measured uniformly because of the contextuality of statistical analysis itself. According to the third prerequisite (contextuality) in CET, only SOC with contributions of many brain regions can ignite consciousness.

The neural complexity *C*_N_ (Tononi et al., 1994) starts with Shannon entropy *H*, defined on a set *X* which can occupy *M* states with probability *p*_i_, satisfying the condition $p=\sum_{i\;=\;1}^{M}p_{i}=1$.


(12) $$H(X)=-\sum_{-i\;=\;1}^{M}p_{i}\log p_{i}$$

Thus, *H*(*X*) = *H_max_* = log *M* if *X* is in equilibrium with $\left(\forall i\right)\;p_{i}=1/M$. On the contrary, *X* has the lowest entropy *H*(*X*) = 0 if *X* will occupy only a single state with *p* = 1. These both comprise the full spectrum of states in which the system *X* could be.

In CET, *X* represents the brain network 𝒩 which can occupy *M* = 2^*N*^ states. Accordingly, the extrema can be applied to the two scenarios in cognitive brain evolution described above: the first with a frog iterating the same jump, and the second with the random number generator where all states from 2^*N*^ configurations are equiprobable. However, it does not propose a quantitative measure of consciousness. To do it, the neural complexity focuses on the structural-functional connectivity of 𝒩. This is mathematically equivalent to the average information exchanged between subsets of a system and the rest of the system, summed over all subset sizes (ideally, beginning with a single neuron). The *C*_𝒩_ can be calculated by mutual information *I* obtained for all possible bipartitions of a system 𝒩 consisting of *N* elements,


(13) $$C_{𝒩}(𝒩)=\sum_{k=1}^{N/2}\left(I({𝒩}_{j}^{k};𝒩-{𝒩}_{j}^{k})\right),$$

where ${Ν}_{j}^{k}$ is the *j*’th bipartition running over all subsets of size *k*, and <· > stands for their average integration. The *I* is defined as


(14) $$I({𝒩}_{j}^{k};𝒩-{𝒩}_{j}^{k})=H({𝒩}_{j}^{k})-H({𝒩}_{j}^{k}|𝒩-{𝒩}_{j}^{k})$$

*C*_𝒩_ is highest when synchronization (integration) and desynchronization (segregation) tendencies are balanced in 𝒩, and lowest under either total synchronization (order) or total desynchronization (disorder) of its elements. In CET, neural complexity should provide a measure for information that was integrated by the brain during a time interval Δ*t*. This displays how well the brain is poised near criticality to gain the optimum information over cognitive brain evolution. Thus, conscious states can emerge with different values of *C*_𝒩_ over the full spectrum of conscious states ranging from UWS to ADHD (as conditioned above).

The measure will reflect the magnitude of the brain’s mental force *at the* given time (Figure 2B). Formally, the level of consciousness in a particular state *S*(*τ*) equals to the neural complexity of its NCC presented by a corresponding vector *x_i_*. Unlike Φ in IIT, consciousness does not depend ontologically on *C*_𝒩_. The distinction can be formalized in the language of CET as follows.


(15) $$\text{IIT}:\;S(\tau )\;\underline{\underline{def}}\underset{\Delta t}{\max}\Phi$$

(16) $$\text{CET}:\;o\;(S(\tau ))=C_{𝒩}(\tau )$$

where *o* is a physical observable of $S(\tau )\;\underline{\underline{def}}\frac{d\psi }{d\tau }$ at given moment τ.

An obvious limitation of *C*_𝒩_ is that the measure reflects the degree of short-term coherency of neural activity irrespectively to the type of cognitive processing recruited there. This can apply to disorders of consciousness (Mateos et al., 2018) such as UWS, but not to mental disorders that depend on long-term coherency of many conscious states over brain dynamics where other complexity (algorithmic) measures are preferable (Aboy et al., 2006; Hager et al., 2017; Murray et al., 2018). On the other hand, it can be proposed that the level of consciousness, typically defined by arousal criteria arranged from coma to full vigilance, is itself irrelevant to cognition. Mashour and Alkire (2013) argue that while humans exhibit the most advanced cognitive capabilities, including language, it is difficult to agree that a fully conscious human has a higher level of consciousness (alertness) than a beast in pursuit of prey.

Nevertheless, it seems undoubted that cognitive processing at the soft level of the brain must somehow affect conscious contents at the psyche level (Yurchenko, 2022). Humans can significantly differ in acquiring information from the same environment while preserving equal sensory abilities. It means that information gain is not affected by their capability to process sensory signals but depends only on how the signals are transformed into cognitive contents provided by brain dynamics (which do not still deviate from criticality). Accordingly, neural complexity should also differ between healthy humans. The difference should be especially significant in states of dementia, where the level of consciousness is preserved despite the dramatic disruption of cognitive abilities. This is a reason why *C*_𝒩_ cannot be a self-sufficient measure of conscious presence. The problem becomes yet more striking if we draw attention to the fly’s brain, which should apparently provide a very low quantity of *C*_𝒩_ compared to the human brain, maybe, even lower than in disorders of consciousness. Should we treat the fly unconscious?

Lamme (2018) recently pointed out that existing theories have missed a “key ingredient” of consciousness. One of its consequences is that many of them while being prone to superdeterminism are at the same time not immune to panpsychism. Thus, on one hand, they endow consciousness with causal power, i.e., free will. On the other hand, they make consciousness a scale-independent phenomenon that can emerge at the atomic or quantum level (Zeki, 2003; Hunt and Schooler, 2019), or that can be inherent to a single neuron or even to a mitochondrion as being ontologically conditioned on Φ (Tononi and Koch, 2015). Therefore, it is not surprising that these theories grant consciousness to future machines (Dehaene et al., 2017). How then should machine consciousness be compatible with free will controlled by hidden deterministic variables *λ*? Also, what would machine consciousness be if each atom already possessed some level of consciousness? CET is immune to both types of panpsychism: the emergence of consciousness is scale-dependent, whereas *C*_𝒩_ is only an epistemic measure of the mental force conditioned on SOC.

Yet, CET asserts that there is no conscious experience without arousal, one of the four principled features (plus SOC) of an arbitrary system should satisfy to be conscious. Recall, these features also comprise: (i) living properties; (ii) cognitive evolution for accumulating knowledge; and (iii) volition not predetermined from the past. In particular, dreams are often taken as evidence that consciousness can be separable from wakefulness (Hobson, 2009; Wamsley, 2013). CET considers dreams differently as the evidence that (passive and discrete) consciousness is complementary to unconscious (active and continuous) cognitive processing, which can persist during REM sleep by involving a parieto-occipital “hot zone” that is typically associated with the cortex-centered NCC (Siclari et al., 2017). CET argues that without activating arousal centers there can be no immediate awareness of something experienced.

As stated, conscious experience is always local in space and time. To be conscious means to be conscious of ourselves here and now while the great gift of imagination can allow us to virtually travel over space and time. Similarly, on retrospection, we can be conscious of what we were doing yesterday or many years ago, but it occurs just now, at the specious present (Varela, 1999). Importantly, we are not conscious while dreaming but we recall what the brain has unconsciously processed just before awakening. Dreams are residual (cortex-centered?) memory the brain can or cannot expose to the psyche level. Indeed, we do not retain dreams every time by awakening.

Yet, we know that all animals, even those endowed with a primitive neutral system, can be anesthetized (Zalucki and van Swinderen, 2016) but there is no “anesthesia” for arbitrary natural or artificial systems like atoms or machines. The arousal centers—even if very different in worms, insects, mollusks, and vertebrates—are that crucial ingredient (Lamme, 2018), without which neither consciousness nor any kind of free will is possible. Thus, consciousness, being inseparable from arousal, could have expanded over the animal kingdom due to the NFVM. Only the level of consciousness would have varied over species depending on the size and architecture of their brain capable of maintaining SOC.

## Discussion

CET adheres to the evidence derived from Liber-type experiments that consciousness is a passive post-factum phenomenon of neural activity with no causal power over brain dynamics. Unfortunately, the free-will experiments have little to say about how the neural activity itself might be free of predetermination in unconscious processing of decision-making. The brain could still remain a deterministic machine (‘t Hooft, 2016; Hossenfelder and Palmer, 2020). Apparently, the only alternative is to adopt quantum indeterminism. The question is then: How to rigorously incorporate the quantum effects into classical stochastic brain dynamics? This problem has produced several quantum proposals in the science of consciousness. First, CET differs from all of them because it does not involve any macroscopic quantum effects that might arguably be exploited by consciousness to speed up the computational power of the brain, and make consciousness non-computable (Fisher, 2015) or even transiently separable from the brain due to closed causal loops (Hameroff and Penrose, 2014).

To CET, quantum indeterminism is not a matter of conscious will, but a matter of evolution of life and origins of consciousness. CET argues that consciousness has no free will but brain dynamics can be free of predetermination in a physically meaningful sense by the minimal use of quantum randomness *via* Beck-Eccles exocytosis. At the same time, Libet-type experiments are all initially cortex-centered by neglecting one obvious neurobiological fact. Wakefulness is a necessary prerequisite of any manipulations with consciousness to detect perception, attention, and cognition as compared to unconscious processing. Neither consciousness nor volition is possible without activating arousal centers.

First, CET rules out conscious will which allegedly might have power over brain dynamics at the soft (computational) level by deciding ahead of time what goal will be achieved (as described above in Alice’s scenario). Second, CET also rejects a statistical (probabilistic) account of volition resulting from noisy neural fluctuations in a stochastic framework (Khalighinejad et al., 2018) because such account of randomness depends ultimately on the state of our knowledge which by itself does not violate determinism. CET then takes the NFVM as a principled argument against superdeterminism, which is the hypothesis that conscious observers cannot act freely or do otherwise than what has been predetermined by deterministic laws of nature. This hypothesis has been suggested with the claim “to remove every single bit of mysticism from quantum theory” (‘t Hooft, 2016) such as a random wavefunction collapse caused by observation or nonlocal (faster than light) correlations between a pair of entangled particles. Nevertheless, contrary to this reasonable claim, superdeterminism would inevitably lead to much more mysterious implications such as “cosmic conspiracies” in Bell-type experiments (Gallicchio et al., 2014) or, more generally, a “designed” universe where conscious beings, controlled by hidden deterministic variables *λ*, do what the universe wants them to do (Yurchenko, 2021b).

Leaving aside these metaphysical issues, CET looks at this problem from an evolutionary perspective on the origins of life and consciousness. Might life have evolved from completely deterministic cause-effect interactions? What advantages would organisms have gained over non-living systems in following the same predetermined ways? Why should consciousness have then evolved from the simplest forms of life? Contrary to the idea that life is a triumph of determinism (Marshall et al., 2017), CET argues that organisms should initially be free of predetermination to have any meaningful difference from non-living systems to evolve from simplest to complex forms of life. In this way, the evolution of the brain would have been driven by the natural selection from primitive neuronal bundles to higher-level neural systems to supply their freedom to maneuver with computational power in an efficient prediction of the environmental events (Brembs, 2011).

This lays a principled division between the cause-effect interactions, reserved for dynamics of non-living systems of any complexity, and the stimulus-response repertoires accessible for all organisms. The repertoires can emerge only from the corresponding neural activity. A lesson we have learnt from biology is that every useful mechanism will be re-used by evolution to promote new more sophisticated mechanisms for adaptive advantages of life. The fast and random reflexes of the simplest organisms could have evolved from quantized micro-events that are abundantly presented in cellular structures of plants and animals (Arndt et al., 2009; Brookes, 2017). The evolution of the brain might have then used those reflexes to build up a neural mechanism like the NFVM in the same way as random (quantum in origin) gene mutations underlie the biological machinery that is responsible for the astonishing variety of species on the Earth. CET places the NFVM into the brainstem, a phylogenetically ancient brain region that comprises various nuclei responsible for executing many vital autonomic functions to maintain homeostasis.

**Figure 3:**
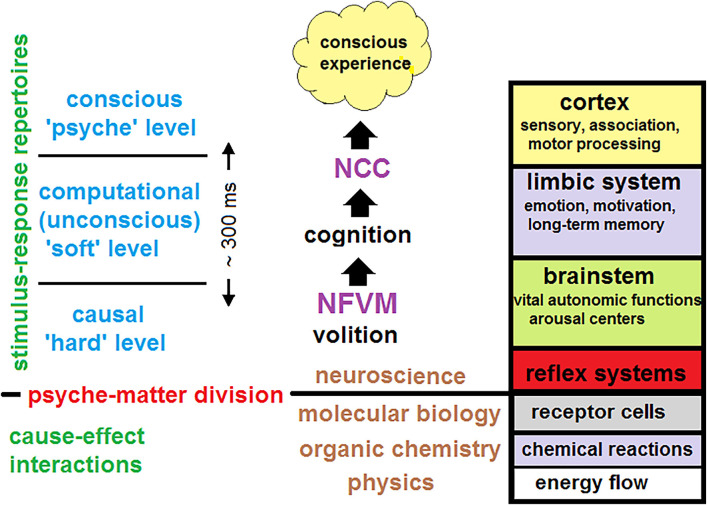
The origins of consciousness. CET starts from the assumption that the brain should have primarily evolved as volitional subsystems of organisms from simplest neural reflexes based on chemoreceptor, magneto- and photoreceptor cells sensitive to quantum effects. At the causal (hard) level, the volitional subsystems should provide a principled psyche-matter division between organisms, exploiting their stimulus-reactions repertoires freely, and non-living systems, governed completely by cause-effects interactions. Placing the NFVM into the brainstem, the oldest brain region that integrates functions of many vital systems and is responsible for arousal and vigilance guarantees that each conscious state will be triggered free of predetermination from the past.

Remarkably, those autonomic functions are typically called “involuntary” in the literature, while voluntary functions are implicitly reserved for conscious volition. The distinction between voluntary and involuntary functions is the main reason for confusion leading to postulating free will. Indeed, unlike reflex-like actions, voluntary actions are by definition those which were initiated consciously. On the other hand, volition must have by definition causal power. Thus, one naturally comes to free will *via* the distinction. In contrast, CET considers all autonomic and unconscious functions just voluntary, i.e., not predetermined from the past even in unicellular organisms placed upon the psyche-matter division between cause-effect interactions and stimulus-reaction repertoires (Figure 3).

This requires a paradigm shift akin to that of the Copernican heliocentrism that had turned upside down the humans’ belief that the Sun revolves around the Earth as they indeed saw it in everyday experience. Similarly, humans believe they have free will to choose the course of future actions under given conditions. Note, CET does not univocally deny this ambiguous formulation. If humans are associated with what their brain does, the brain can indeed make a choice that had not been predetermined from the past. This scenario is possible on the condition that the choice is causally triggered at the hard level by the NFVM in the arousal centers of the brainstem. The quantized micro-event will then be amplified *via* neural scale-free avalanches and cognitively modulated at the soft level across thalamocortical systems in unconscious ways, and exposed ultimately to the psyche level.

On the contrary, CET completely denies the assumption that humans are associated with what their consciousness might do to their brain (and their body) by intervening in neural activity at the hard level. In the stream, particular conscious states are what the brain has periodically generated at critical points of brain dynamics as it explores its attractor space (yet bombarded by sensory signals). The only distinction between involuntary and voluntary actions depends on how the brain processes decision-making, which, in turn, depends on the cognitive complexity of a task. Simple or intermediate tasks as those modeled in multilayer predictive processing (Pezzulo et al., 2018) will be computed unconsciously. Only the ultimate decisions of complex tasks will be exposed to the psyche level as particular conscious states (which can in principle be measured as the brain’s mental force at a given moment).

Take for example breathing and heartbeat, both occurring without necessarily requiring consciousness. We can nonetheless stop breathing for some time by focusing our attention on this autonomic process. Advocates of free will take this as an evidence of the causal power of consciousness. They concede, however, that we are still unable to stop the heartbeat. Importantly, in CET, the stream of “pulsating” consciousness is itself like a heartbeat being every time initiated by the NFVM from arousal centers. At the same time, meditators trained in yoga practice are believed to be able to stop their heartbeat too (Krygier et al., 2013; Vinay et al., 2016). Does it mean that their free will is more powerful than that of ordinary humans? If so, does it also mean that free will weakens in humans with mental diseases such as obsessive-compulsive disorder or schizophrenia? Or is it all a matter of the cognitive effort and the corresponding state of the thalamocortical systems?

CET considers wrong saying that “A trained consciousness can control involuntary heartbeat at its will” or “A cognitively disrupted consciousness cannot well control even voluntary behavior at its will”. In both cases, the brain possesses consciousness as its mental force generated at a given moment of time. This is what produces the illusion of free will as if consciousness itself (like a homunculus) “strikes” the force from the brain to command future actions. Hence, all conscious states in the stream are already causally driven by the brain like breathing and heartbeat. They all can be called voluntary on the authority of the brain. For instance, the experience of “acting involuntarily” like suddenly reaching out to catch a falling object, or reflexively removing one’s body from a hot object may arise because such relatively simple actions can be produced at the hard level before its stimulus had been exposed to consciousness at the psyche level.

CET argues that the stream of consciousness (*way of being*) is initially NFVM-driven from arousal centers, and can be then derived—in any meaningful (formal or informal) sense—only from cognitive brain evolution (*way of knowing*). The brain continuously accumulates information to make an embodied choice, whereas unitary conscious states appear instantaneously at critical points of brain dynamics as ultimate decisions (snapshots) of unconscious action-oriented cognitive processing (Engel et al., 2013). The stream *S*(*τ*) cannot go on, though, it can persist like a freeze-frame on a TV screen if cognitive brain evolution stops and working memory has nothing to update, as it occurs in patients with UWS when brain dynamics depart from criticality (Tagliazucchi et al., 2016; Lee et al., 2019; Huang et al., 2020). Indeed, patients who recovered after UWS have no memories of their time in rehabilitation as if the stream was “frozen” all the time (Gosseries et al., 2014).

While the emergence of consciousness from brain dynamics is commonly accepted, many authors still ascribe to consciousness a special biological function and active role for selective attention, operant learning, and goal-directed behavior as if evolution had equipped the brain with consciousness just to perform complex volitional actions, which might not be performed unconsciously (Pennartz et al., 2019; Feinberg and Mallatt, 2020; Rolls, 2020). Again, CET does not univocally deny this conjecture. On one hand, consciousness has never been the goal of evolution but it has developed gradually from neural activities of organisms as a global byproduct of their volitional-cognitive mechanisms, however, with no causal power in brain dynamics. On the other hand, conscious states are exposed to the psyche level as ultimate decisions of complex cognitive tasks processed at that moment.

The laws of Nature make consciousness a necessary emergent phenomenon of brain dynamics in the same way as some quantity of H_2_O molecules (but not one or two) placed together will necessarily produce water. Turn now water into a neuronal substance, add SOC, a volitional (quantum in origin) mechanism, arousal centers, and learning for accumulating knowledge: these ingredients will spontaneously produce consciousness.

Does it mean that the hard problem of consciousness can be solved? Generally, nothing in CET prevents one from assuming that future AI systems endowed with these neuromorphic properties may one day become conscious. Nevertheless, these machines would be non-deterministic and, hence, as unpredictable as humans are. In other words, we might not circumvent Nature in order to create conscious automata like selfless lackeys. Moreover, such conscious and selfish machines might not be immune to disorders and psychoses such as “machine schizophrenia” or “machine depression.” CET argues that the hard problem as it is related to the search for NCC (Crick and Koch, 2003) can be better understood by linking it to the secured privacy of a particular consciousness. If a machine might know what it is like to be Alice, the uniqueness of her subjective experience would be uncovered and in principle copyable. Many machines might have the same qualia. In this sense, subjective qualia emerging phenomenally at the psyche level of Alice’s brain are a non-eliminable part of her (and only her) consciousness.

In a conventional view, humans possess free will due to conscious deliberation. The view seems to be maintained by plenty of experimental evidence showing that, in contrast to reflex (autonomic) actions, the cortical function is essential for volitional control of self-initiated actions (Libet, 1985; Haggard, 2008; Fried et al., 2011). However, an obvious thing is neglected in those findings: before executing conscious control in sensorimotor systems of the cortex, the brain should already maintain conscious experience due to arousal centers. The cortex-centered account of free will in Libet-type experiments loses its validity the moment one realizes that no kind of volition can be ascribed to a subject in coma after brainstem lesions (Laureys et al., 2004).

CET proposes a paradigm shift based on an evolutionary and physically rigorous perspective, contrary to the conventional view: humans possess consciousness due to a volitional (Bell-certified) mechanism. While rapid and random reflexes represent the most primitive preconscious form of volitional motor integration, consciousness is the highest phenomenon of global integration. In CET, consciousness is like a river buoy fluctuating on the surface of water regardless of its depth. On the other hand, precisely because of its representative and “superficial” nature in brain dynamics, the stream of consciousness is the marker the brain has exposed to its environment (including external observers) in a meaningful sense.

## Conclusion

Assuming consciousness to be active in brain dynamics entails three intrinsically linked corollaries: it should be: (i) *temporally continuous* to implement its own (ii) *conscious processing* in parallel with unconscious processing. Brain dynamics would be therefore divided into two interdependent parts, a “highway” for conscious processing and an “underground” for unconscious processing, each requiring some (iii) *neural* *correlates* (workplace) for executing their causal function in the brain. Rejecting the active role of consciousness challenges these corollaries as well. First of all, CET operates exclusively on the stream of consciousness as a discrete chain of momentary states ignited at/near criticality without assuming any separate highway for conscious processing. Accordingly, what we experience as a state of perceptual presence has no special NCC in the brain but spreads over many brain regions as those currently involved in volition and cognition.

Moreover, consciousness is not only the phenomenal experience of something; it implies one’s self-awareness. Perhaps, the only biological function assigned to consciousness by evolution is self-awareness, i.e., the ability not only to be but also to have *the sense of being*. Consciousness gives the meaning of life to organisms—not philosophically but biologically: it is the evolutionary reward of the brain for making its work, over which the variety of all sensations and all values of being alive unfold. Life could not have succeeded on Earth if organisms did not appreciate these values.

## Data Availability Statement

The original contributions presented in the study are included in the article, further inquiries can be directed to the corresponding author.

## Author Contributions

The author confirms being the sole contributor of this work and has approved it for publication.

## Funding

I would like to thank Frontiers for funding the APC of this article.

## Footnotes

^1^Here, the mechanism is called “neurophysiological” to emphasize that CET has nothing to do with any kind of mind-brain dualism or psyche-matter complementarity, advocated by prominent scientists such as von Neumann, Pauli, Wheeler, Eccles, and Popper. CET does not also admit downward (top-down) causation, a covert version of dualism that might endow consciousness with causal power over the brain (Hoel et al., 2013; Mediano et al., 2022). Yet, the word “free” implies that causal power of volition must necessarily be free of predetermination. That is, CET does not adopt a compatibilist account of free will.

^2^Traditionally, affective neuroscience holds that activation of evolutionary ancient subcortical regions is both necessary and sufficient for affective experience, whereas cognitive neuroscience argues that subcortical processes are necessary but not sufficient even for primitive experience. Remarkably, many theories of consciousness are conceived in the context of cognitive neuroscience to account—explicitly or implicitly—for active consciousness, i.e., free will that should be cortex-centered. In contrast, CET discards the active role of consciousness and maintains the claim of affective neuroscience.

^3^Recall, in CET, discrete conscious states are exposed to the psyche level as the ultimate decision of cognitive (predictive and unconscious) processing at the soft level (Yurchenko, 2022). These decisions form the stream *S*(*τ*) in the physical time continuum with temporal resolution by Δ*t*. For example, if placed between two almost identical images, excepting a tiny detail, consciousness perceives each as a whole image (gestalt) per state, gaining then an insight into the difference. The whole enterprise of making animation movies relies on this brain’s ability to capture very similar visual scenes (discrete frames about 24 per second) and to make a difference by comparing them, perceived then consciously over stream *S*(τ) as a dynamical picture of natural motion in time. This is the reason why the illusion of active consciousness and the illusion of continuous consciousness converge and make the use of the term “conscious processing” legitimate in the literature.
